# MSA Class Logos: a web server for automated sequence logo generation for user-defined sequence classes based on one multiple sequence alignment

**DOI:** 10.1093/bioadv/vbaf217

**Published:** 2025-09-11

**Authors:** Mauricio F González-Reyes, Fabio Durán-Verdugo, Alejandro Valdés-Jiménez, Janin Riedelsberger

**Affiliations:** Escuela de Ingeniería Civil en Bioinformática, Facultad de Ingeniería, Campus Talca, Universidad de Talca, Talca 3460000, Chile; Escuela de Ingeniería Civil en Bioinformática, Facultad de Ingeniería, Campus Talca, Universidad de Talca, Talca 3460000, Chile; Departamento de Sistemas de Información, Universidad del Bío-Bío, Concepción 4051381, Chile; Centro de Bioinformática, Simulación y Modelado (CBSM), Facultad de Ingeniería, Campus Talca, Universidad de Talca, Talca 3460000, Chile

## Abstract

**Summary:**

Sequence logos are a common way to visually represent amino acid frequencies and conserved sequence patterns of multiple sequence alignments (MSAs). MSA Class Logos is a free web server that enables users to browse and explore sequence logos of several sequence groups or classes simultaneously online, and to download amino acid frequencies for the entire MSA or the sequence classes. Amino acid frequencies can be downloaded in tabular or graphical form for further offline analysis. The core feature of MSA Class Logos is the user’s ability to group amino acid sequences into classes or subgroups, generate sequence logos for each class, and compare the clearly arranged logos online. Sequence logos for the entire MSA and user-defined classes are generated in separate tabs and can be explored conveniently in parallel. Class-specific sequence logos facilitate the identification of class-specific conserved residues unique in evolutionary distant or functionally differing protein groups. Results can be explored visually directly on the web page or downloaded as tables in CSV format. Additionally, segments of each sequence logo can be downloaded in SVG format. MSA Class Logos is primarily intended for investigators without programming and scripting skills to ease detailed sequence analysis.

**Availability and implementation:**

MSA Class Logos and further documentation is available at https://msaclasslogos.appsbio.utalca.cl.

## 1 Introduction

Sequence logos were introduced in 1990 by Schneider and Stephens and are a common way to visualize conserved sequence regions and sequence patterns (Schneider and Stephens 1990). Logos are graphical representations of amino or nucleic acid frequencies where occurrences translate into letter sizes. Multiple sequence alignments (MSAs) are the basis for sequence logos generation, and several online tools are available to generate them. A widely used tool for sequence logo generation is WebLogo3, which was developed based on the original software MakeLogo by Schneider and Stephens (Crooks *et al.* 2004). Many derivates have been designed since, including the integration of statistical parameters or k-mer probabilities like in LogOddsLogo or kpLogo, to name just a few (Yu *et al.* 2015, Wu and Bartel 2017).

Interestingly, the web tool Two Sample Logo enables the comparison of two MSAs and displays graphically statistically significant differences between them (Vacic *et al.* 2006). In addition, flexible sequence logo-generating software and libraries, such as ggseqlogo for R or Logomaker for Python, are available to generate publication-ready sequence logos (Wagih 2017, Tareen and Kinney 2020). Despite the plethora of existing software, to our knowledge, no available tool permits the parallel interactive exploration of several sequence logos based on the input of a single MSA containing the complete set of sequences to analyze. Also, many of the customizable tools, like Python or R libraries, require certain programming or scripting skills, which some scientists do not have. Here, we present MSA Class Logos, a web-based online tool allowing the selection of classes or sequence groups within one MSA provided by the user. Sequence logos are generated automatically for the entire MSA and each defined sequence class, which can be explored and compared quickly and flexibly online. Selected information can additionally be downloaded for further offline analyses.

## 2 Implementation

MSA Class Logos was implemented using the Python programming language, and the web application was generated using the Flask Python-based web framework (Flask 2024). The software analyzes protein sequences and generates sequence logos based on MSAs provided by the user. The main library used for data processing is Biopython, which provides valuable tools for computational biology applications and is used to handle all workflows related to protein sequence processing (Cock *et al.* 2009). JavaScript was used to allow for a fluid and responsive visualization of sequence logos. For the export of sequence logo images, WebLogo was integrated, a robust tool widely used in the bioinformatics community (Crooks *et al.* 2004). For efficient visual comparison of several sequence logo classes, the Python-based open source code svg_stack was used to export identical sequence regions for all sequence classes in a single image (svg_stack 2024). Results are stored on the server for one month before their deletion.

The workflow of MSA Class Logos is as follows: (i) the user provides an MSA in FASTA format and optionally a CSV file containing each sequence’s assignation to a user-defined class. If no CSV file is specified, sequences can be assigned to user-defined classes directly on the web application. (ii) The software processes each sequence and organizes it into a specific class according to the CSV file provided by the user or the classes manually created on the web application. (iii) Sequences are processed to generate interactive sequence logos showing amino acid frequencies for both the complete MSA and each defined sequence class. In addition, consensus sequences for each sequence class are determined.

Sequence logos are represented in two ways: (i) sequence logos for the complete MSA (All Sequences), and (ii) sequence logos for each pre-defined sequence class (Class Specific Logos). Both views are displayed in separate tabs, enabling the user to analyze them in parallel. Results are visually explorable online and also downloadable. Variable output tables of amino acid frequencies per MSA position are available for download in CSV format. See Section 3 for further details.

## 3 Results

**Figure 1.**
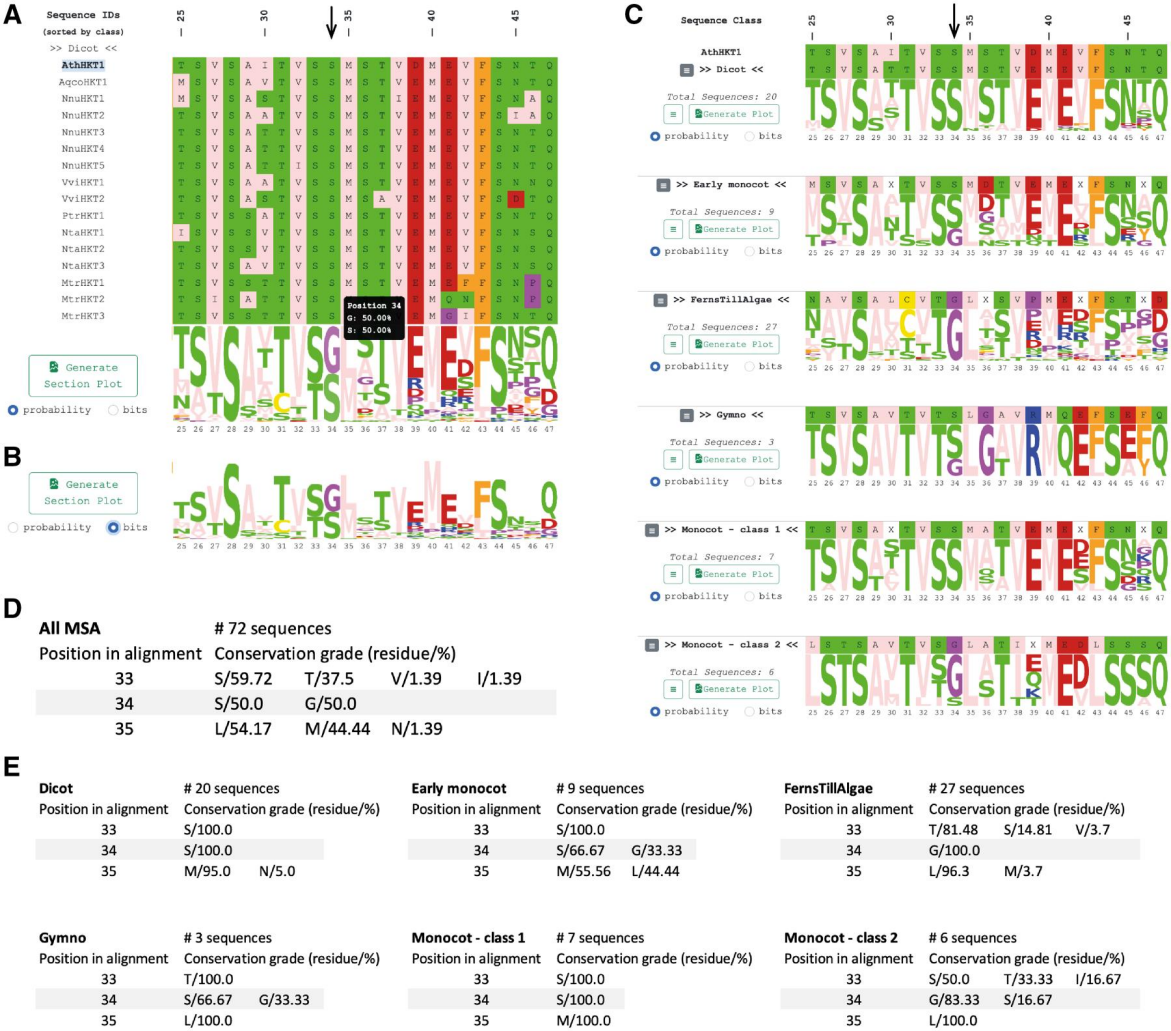
Result views provided by MSA Class Logos. (A) Sequence logo for the complete MSA provided in the “All Sequences” tab. Per default, the sequence logo is displayed in the probability view. (B) The sequence logo for the complete MSA in bits view. (C) Sequence logos for each user-defined sequence class provided in the “Class Specific Logos” tab. (D) Exemplary output from the downloadable CSV file showing amino acid frequencies for the complete MSA (“Amino acid frequencies complete alignment”). For clarity, only positions 33 to 35 are shown. (E) Exemplary output from the downloadable CSV file showing amino acid frequencies for MSAs of user-defined sequence classes (“Amino acid frequencies per class”). Also here, only positions 33 to 35 are shown.

Dependent on the sequence logo and the view selected, the user can extract different information from the data. The “All Sequences” tab visualizes the entire MSA with sequences sorted according to the classes defined by the user (Fig. 1A). Below the sequences is the sequence logo located. Amino acids are colored according to the Zappo coloring scheme highlighting the physicochemical properties of amino acids. For convenience, the color scheme is detailed on the top left. The height of each position depends on the frequency with which an amino acid occurs in the MSA. Two sequence logo views are implemented—probability and bits. The probability view displays each amino acid according to the frequency it has in a certain position in the MSA. Gaps are considered in this calculation since they contribute to characterizing a position in the MSA, which is why the one-letter amino acid codes in some columns do not reach the maximum height of 100% (Fig. 1A) (Pérez-Bercoff *et al.* 2006). The bit view, on the other hand, considers the entropy of the amino acid distribution in comparison to the maximum possible entropy at a given position as described and defined by Schneider and Stephens and by Crooks and colleagues (Schneider and Stephens 1990, Crooks *et al.* 2004) (Fig. 1B). Moving the cursor above a position in the sequence logo shows the amino acid frequencies for this specific position.

Sequence logos for the defined classes are displayed in the “Class Specific Logos” tab (Fig. 1C). Here, the previously selected reference sequence is displayed on top for reference purposes only. Below, each sequence class is represented by its proper sequence logo and a consensus sequence. The consensus sequences display the most frequent amino acid at a position. Should two or more amino acids share the highest frequency, this position is marked with an X. On the left, the name of each sequence class and the number of sequences defined for this class are shown.

The benefit of comparing sequence logos of a subset of sequences (sequence classes) versus the sequence logos of an entire MSA becomes visible by examining position 34 of the example MSA in more detail. Figure 1A and B show at position 34 of the MSA the presence of either a serine or a glycine. Each of the amino acids appears in 50% of the analyzed sequences. Examining position 34 in the “Class Specific Logos” tab reveals that some groups express exclusively a serine residue (e.g. Dicots class), others exclusively a glycine residue (e.g. FernsTillAlgae class), and other classes allow the presence of serine or glycine like the Early monocot class (Fig. 1C). The presented MSA is an extract of a collection of plant HKT ion channels, which have been analyzed and reviewed recently (Riedelsberger *et al.* 2021). Two classes of HKT proteins are known, which express differences in ion selectivity attributed to a polymorphism in the first pore loop. The sodium-selective HKT channels mostly contain a serine in the first pore loop, and sodium and potassium-selective channels a glycine. Position 34 from our example MSA corresponds to the described polymorphism. MSA Class Logos visualizes clearly that sodium-selective HKT proteins from dicotyledons (Dicots class) and class 1 monocotyledons (Monocot—class 1) express a serine while sodium and potassium-selective class 2 monocotyledons (Monocot—class 2) predominantly express a glycine. The “Class Specific Logos” tab allows for easy identification of polymorphisms that discriminate sequence classes and may reveal the molecular basis for class-specific features.

In addition to visually inspecting the sequence logos on the web application, amino acid frequencies can be downloaded in CSV format and processed offline. Several result outputs are pre-defined. The files “Amino acid frequencies complete alignment” and “Amino acid frequencies per class” contain the frequencies for all positions based on the entire alignment or separated per class (Fig. 1D and E). These files cover the whole length of the MSA. Other result files with a percentage specified in their name contain only a subset of positions. For example, the file “Positions 100% conserved” contains only the positions with amino acids showing a 100% frequency, in other words, 100% conserved positions. The file “Positions ≥90% conserved” contains all positions with amino acids conserved in 90% or more sequences, therefore, with frequencies above 90%. Files with lower percentages in their name apply respective lower filters. Additionally, selected regions of the sequence logos can be exported in SVG format in probability or bits view from both tabs. In the “Class Specific Logos” tab, sequence logos can be exported for one specific class (“Generate Plot” on the left) or for all classes at once (“Generate Plot All Classes” on top). Furthermore, selected sequence classes can be hidden from the “Class Specific Logos” tab by clicking on the buttons with three horizontal lines on the left. The “Table Reset” button on the top restores all hidden classes. Finally, the MSA to be analyzed on this web application may be a collection of sequences of the same family or joined sequences of different families where each family will be defined as a class to compare logos of two or more families directly.

## 4 Conclusion

MSA Class Logos presents a flexible tool for analyzing MSAs in more detail. Subsets of the MSA (classes) can be defined to generate individual sequence logos that are displayed and arranged one below the other and can easily be screened by scrolling through them. The organization of the results in two tabs provides a flexible way of exploring intriguing areas of the MSA quickly, extracting specific information, and putting observations in context. As the example in the result section showed, MSA Class Logos allows the straightforward visualization of class-specific differences in amino acid sequences that may be not obvious in big MSAs. MSA Class Logos reduces the valuable time of investigators and minimizes errors due to the automated generation of several sequence logos in parallel, which allows to concentrate focus and energy on the scientific aspect of sequence analysis. Finally, no programming skills are necessary to obtain and analyze results.

## Data Availability

No new data were generated or analysed in support of this research.
